# Editorial: Enhancing Drug Delivery and Tumor Penetration

**DOI:** 10.3389/fonc.2021.775001

**Published:** 2021-10-29

**Authors:** Yuxia Tang, Meihua Yu, Xiaodan Su, Zhaogang Teng, Shouju Wang

**Affiliations:** ^1^ Laboratory of Molecular Imaging, Department of Radiology, The First Affiliated Hospital of Nanjing Medical University, Nanjing, China; ^2^ Diamantina Institute, University of Queensland, Woolloongabba, QLD, Australia; ^3^ Key Laboratory for Organic Electronics & Information Displays and Institute of Advanced Materials, Nanjing University of Posts and Telecommunications, Nanjing, China

**Keywords:** drug delivery, tumor penetration, nanocarrier, biological barrier, optimal design

Chemotherapy is vital for cancer treatment; however, most drugs used in the treatment are hydrophobic and difficult to administer. Nano-drug delivery systems (NDDS) have been widely used to transport drugs to the target site because the system improves drug bioavailability and prevents drug degradation. However, intravenously administered nanoparticles face various barriers in the body. Most nanoparticles circulating in the blood are removed by the reticuloendothelial system (RES) in the liver and spleen. Moreover, NDDS extravasation leads to drugs being distributed in the tumoral peripheral vasculature instead of penetrating the tumor.

Recently, several strategies, including optimizing the physicochemical properties of nanocarriers and regulating tumor microenvironments, have been investigated to lengthen the presence of NDDS in the blood and enhance the tumor penetrating ability of NDDS. The most common method to prolong the circulation time of NDDS is to graft polyethylene glycol onto the nanocarriers. The hydrated layer formed by the stealth of polyethylene glycol can reduce the clearance by RES. To enhance tumor targeting, researchers have optimized the surface, size, shape, and zeta potential of nanocarriers in NDDS. The ideal NDDS design may be complex based on known nano-bio interactions is complex *in vitro* and *in vivo*. Many studies have elucidated the impact of nanocarrier properties in tumor delivery. Various stimuli-responsive nanocarriers have recently been developed due to the different pH values and redox states of tumor tissues. These smart NDDS do not allow drug release during blood circulation but promotes tumor targeting. Moreover, deep tumor penetration could be achieved by regulating the tumor microenvironment through interstitial fluid pressure reduction, dense stroma depletion, and nano-tumor interaction regulation. This Research Topic includes two review articles and five original research articles on drug delivery and enhanced tumor penetration.

Focusing on evaluating the efficacy of drug delivery, Kashkooli et al. proposed a mathematical model of drug transport to predict chemotherapeutic drug delivery, either in free form or encapsulated in nanoparticles. The study of new drug targets and drug structures is essential for drug distribution in tumors. Liang et al. summarized the recent progress in compounds containing 1,2,3-triazole to treat lung cancer and discussed their structure-activity relationship and the mechanisms of action. In the review, the authors provided a rational design of derivatives containing 1,2,3-triazole that could lead to new anticancer drugs with great efficacy against various lung cancers. Liu et al. demonstrated that downregulation of Nrf2 could inhibit invasion, metastasis, and angiogenesis of hepatoma, providing a potential therapy for hepatocellular carcinoma. Chen et al. constructed a nanodrug with improved tumor targeting by using a doxorubicin-conjugated polymer that responds to light and self-assembles to form polymeric micelles in water. Alhakamy et al. improved the anticancer efficacy of 2-methoxy estradiol in prostate cancer by using a hydrophobic micelle core formulated with Phospholipon 90G and d-α-tocopheryl polyethylene glycol succinate (TPGS). The formulation was optimized using the Box-Behnken statistical design and Statgraphics software to standardize TPGS and phospholipid percentages and to obtain the smallest particle size. Cheng et al. reported a combined drug administration method to improve efficacy in the treatment of high-risk non-muscle-invasive bladder cancer through intra-arterial chemotherapy combined with intravesical chemotherapy. Because the regulation of tumor stroma is a critical strategy to enhance drug penetration, nanozymes, which are effective in the depletion of tumor microenvironments, should be studied further. Liao et al. reviewed the latest advances in nanozymes in tumor and tumor microenvironment diagnosis, therapy, and theranostics.

NDDS have developed rapidly, that they now overcome biological barriers and facilitate effective treatment with reduced adverse effects. Many reports have discussed the recent advances in drug delivery and penetration; however, new NDDS designs and tumor stromal regulation are yet to be fully addressed It is necessary to analyze the properties of NDDS, investigate the behaviors of nanocarriers, and understand the various barriers during tumor penetration. The editors hope that the published articles on this Research Topic will inspire future work for a deeper understanding of drug delivery and tumor penetration.

## Author Contributions

All authors listed have made a substantial, direct and intellectual contribution to the work and approved it for publication.

## Funding

This work received funding from the National Natural Science Foundation of China (Nos 82022034 and 81871420) and Jiangsu Province Natural Science Foundation of China (No. BK20200032).

## Conflict of Interest

The authors declare that the research was conducted in the absence of any commercial or financial relationships that could be construed as a potential conflict of interest.

## Publisher’s Note

All claims expressed in this article are solely those of the authors and do not necessarily represent those of their affiliated organizations, or those of the publisher, the editors and the reviewers. Any product that may be evaluated in this article, or claim that may be made by its manufacturer, is not guaranteed or endorsed by the publisher.

